# Somatic *APC* mosaicism and oligogenic inheritance in genetically unsolved colorectal adenomatous polyposis patients

**DOI:** 10.1038/s41431-017-0086-y

**Published:** 2018-01-24

**Authors:** Michele Ciavarella, Sara Miccoli, Anna Prossomariti, Tommaso Pippucci, Elena Bonora, Francesco Buscherini, Flavia Palombo, Roberta Zuntini, Tiziana Balbi, Claudio Ceccarelli, Franco Bazzoli, Luigi Ricciardiello, Daniela Turchetti, Giulia Piazzi

**Affiliations:** 1 Medical Genetics Unit, S.Orsola-Malpighi Hospital, University of Bologna, Bologna, Italy; 2 Center for Studies on Hereditary Cancer, Department of Medical and Surgical Sciences, University of Bologna, Bologna, Italy; 3 Department of Medical and Surgical Sciences, University of Bologna, Bologna, Italy; 4 Center for Applied Biomedical Research (CRBA), S.Orsola-Malpighi Hospital, University of Bologna, Bologna, Italy; 5 Pathology Unit, S. Orsola-Malpighi Hospital, University of Bologna, Bologna, Italy; 6 Department of Experimental, Diagnostic and Specialty Medicine, University of Bologna, Bologna, Italy

## Abstract

Germline variants in the *APC* gene cause familial adenomatous polyposis. Inherited variants in *MutYH, POLE*, *POLD1, NTHL1*, and *MSH3* genes and somatic *APC* mosaicism have been reported as alternative causes of polyposis. However, ~30–50% of cases of polyposis remain genetically unsolved. Thus, the aim of this study was to investigate the genetic causes of unexplained adenomatous polyposis. Eight sporadic cases with >20 adenomatous polyps by 35 years of age or >50 adenomatous polyps by 55 years of age, and no causative germline variants in *APC* and/or MutYH, were enrolled from a cohort of 56 subjects with adenomatous colorectal polyposis. *APC* gene mosaicism was investigated on DNA from colonic adenomas by Sanger sequencing or Whole Exome Sequencing (WES). Mosaicism extension to other tissues (peripheral blood, saliva, hair follicles) was evaluated using Sanger sequencing and/or digital PCR. *APC* second hit was investigated in adenomas from mosaic patients. WES was performed on DNA from peripheral blood to identify additional polyposis candidate variants. We identified *APC* mosaicism in 50% of patients. In three cases mosaicism was restricted to the colon, while in one it also extended to the duodenum and saliva. One patient without *APC* mosaicism, carrying an *APC* in-frame deletion of uncertain significance, was found to harbor rare germline variants in *OGG1*, *POLQ*, and *EXO1* genes. In conclusion, our restrictive selection criteria improved the detection of mosaic *APC* patients. In addition, we showed for the first time that an oligogenic inheritance of rare variants might have a cooperative role in sporadic colorectal polyposis onset.

## Introduction

Approximately 1% of all the colorectal cancer (CRC) cases are due to familial adenomatous polyposis (FAP), an autosomal dominant CRC predisposition syndrome with a penetrance close to 100% [1]. The classic FAP phenotype is characterized by the development of multiple (hundreds to thousands) colonic adenomatous polyps at early age [2]. Moreover, FAP patients frequently develop extra-colonic manifestations, including upper gastrointestinal and desmoids tumors, mandibular osteomas, and hypertrophic pigmentary lesions of the retina [3]. Typically, FAP arises on heterozygous germline variants in the Adenomatous Polyposis Coli (*APC*) tumor-suppressor gene located on chromosome region 5q21–22 [4]. The *APC* gene encodes for a protein that is critically involved in the canonical Wnt signaling pathway, the activation of which leads to β-catenin nuclear translocation and intestinal epithelium hyperproliferation [5]. Most inactivating *APC* germline variants are frameshift or nonsense; in addition, the *APC* gene could be inactivated through promoter hypermethylation or large deletions [6, 7]. A high frequency of de novo* APC* variants (10–25%), generally affecting the “mutation cluster region”(MCR; codons 1286–1513) [6], has been reported in FAP patients [8, 9]. Moreover, somatic mosaicism in the *APC* gene has been described in a small subset of FAP cases [10–12].

Biallelic inactivation of the MutY homolog (*MutYH*) gene causes an autosomal recessive form of polyposis, characterized by the development of few adenomas and progression to CRC at an older age than classic FAP [13]. Variants in this gene were found in ~20% of cases with attenuated polyposis [14]. The *MutYH* gene, located on chromosome region 1p34.3–1p32.1, encodes a protein involved in the Base Excision Repair (BER) pathway that prevents DNA damage induced by 8-oxo-7, 8-dihydro-2′-deoxyguanosine [13]. In addition, rare forms of colorectal polyposis are caused by variants in *POLE*, *POLD1*, and *NHTL1* genes [15–17]. *POLE* and *POLD1* genes encode for the main catalytic and proofreading subunits of polymerase ε and δ enzyme complex, critically involved in DNA replication fidelity [18]. *NTHL1* gene encodes for a member of the BER pathway that removes oxidized pyrimidines and ring-opened purines [19]. Recently, biallelic germline variants in the *MSH3* gene, a member of the DNA mismatch repair (MMR) system, has been reported as an additional genetic cause of colorectal polyposis [20].

However, to date, in ~30–50% of cases, the genetic defect responsible for the onset of colorectal polyposis remains unknown [21], leading to uncertainties in establishing proper clinical management and risk for relatives. Thus, the main goal of this study was to identify the genetic defect in a set of adenomatous polyposis patients with no germline variants in known predisposing genes, aiming to provide an appropriate diagnosis and treatment. Through a rigorous selection of candidate patients, we were able to identify *APC* mosaicism as the main cause of colorectal polyposis in 50% of the enrolled patients (4 out of 8), and to define a new oligogenic inheritance model that could explain an *APC*-independent polyposis in one patient.

## Methods

### Patients and data collection

**Fig. 1:**
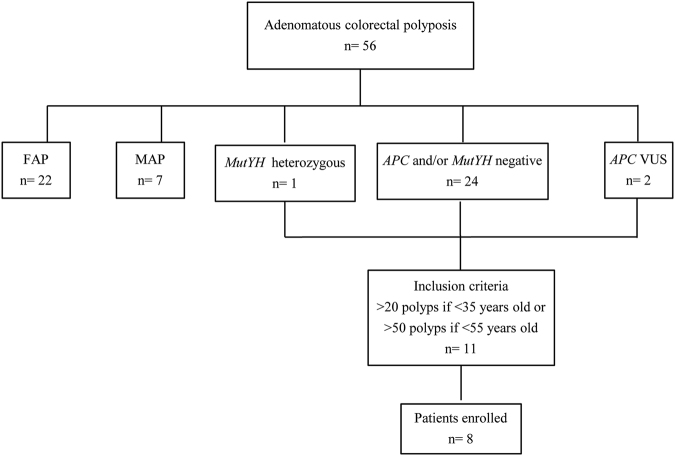
Flowchart of the patients’ mutational screening, selection, and enrollment

From January 2004 to March 2016, 56 patients with a clinical and histological diagnosis of adenomatous colorectal polyposis underwent genetic counseling at the Familial Colorectal Cancer Clinic of the Sant’Orsola-Malpighi Hospital (Bologna, Italy). As shown in Fig. 1, 29 (51.8%) patients were found to carry a causative variant in *APC* (*n* = 22) or *MutYH* (*n* = 7) genes. Among patients with no conclusive genetic diagnosis, those with 20 or more adenomatous polyps by 35 years of age or with 50 or more adenomatous polyps by 55 years of age were considered eligible for this study. Polyps were histologically characterized by an expert pathologist (T.B.). Patients with polyps histologically different from adenomas were excluded. Of the 11 eligible patients, 8 accepted to participate and provided their informed written consent. None of the enrolled patients had a family history of adenomatous polyposis. Clinico-pathological characteristics of enrolled patients are reported in Table 1. For all patients, Formalin-Fixed Paraffin-Embedded (FFPE) tissues (adenomatous polyps and normal mucosa) from different endoscopic sessions and a blood sample were obtained. For five patients (P1–P4, P8), fresh adenomatous polyps (<5 mm) and normal colonic mucosa samples were also collected during colonoscopy, and stored in RNAlater® (Thermo Fisher Scientific, MA, USA) until DNA extraction. For patients with *APC* mosaicism, hair follicles and saliva samples were also collected. When possible, peripheral blood samples from probands’ parents and/or siblings (Table 1) were obtained. Peripheral blood samples from two healthy subjects were obtained and DNA was used as reference samples for some analyses. The study was conducted in accordance with the Declaration of Helsinki and was approved by the Ethics Committee of the S.Orsola-Malpighi Hospital, Bologna, Italy.

**Table 1:** Clinico-pathological characteristics of enrolled patients (*n* = 8)

Patient	Gender	Age^a^ (Years)	N Polyps	Additional clinical manifestations	Parents/siblings^b^
P1	F	32	>50	No	Yes
P2	M	24	>100	No	Yes
P3	F	47	>150	Duodenal adenomas; Bilateral sensorineural hearing loss; Diabetes mellitus type II	Yes
P4	M	23	>20	No	Yes
P5	M	29	>20	Proctocolectomy 2 synchronous adenocarcinomas	Yes
P6	M	54	>50	Proctocolectomy 2 synchronous adenocarcinomas	No
P7	M	55	>50	Urothelial cancer; Total colectomy	No
P8	F	40	>50	Sub-total colectomy	Yes

^a^Age at diagnosis

^b^Available blood samples

### DNA extraction

DNA from peripheral blood, saliva, and hair follicles was isolated using the QIAmp^®^ Blood Mini Kit, although DNA from fresh colonic tissues was obtained using the AllPrep^®^ DNA/RNA/Protein Mini kit (Qiagen, Milan, Italy) according to the manufacturer’s protocols. DNA from FFPE tissues was extracted using the Maxwell^®^16 FFPE Plus LEV DNA Purification kit (Promega, Milan, Italy) after macrodissection. DNA concentration was measured using the Nanodrop 1000 spectrophotometer (Thermo Scientific, USA).

### *APC* variant screening and mosaicism detection

For *APC* variant screening, the entire coding region of the *APC* gene (RefSeq NM_000038.5), as well as promoters 1A (RefSeq U02509.1) and 1B (RefSeq D13981.1), were sequenced by Sanger sequencing on DNA extracted from one fresh adenomatous polyp, except for patient P1, where two polyps where analyzed. Any *APC* variant identified in a polyp was checked on FFPE DNA samples of at least four independent adenomatous polyps and two samples of normal mucosa. Primer sequences and annealing temperatures are reported in Supplementary Table S1A. A condition of mosaicism was assumed if the same pathogenic variant was present in at least four independent FFPE adenomatous polyps.

### *APC* mosaicism extension evaluation

To investigate the *APC* mosaicism extension within the three germ layers, DNA extracted from fresh and available FFPE colonic tissues (endoderm), peripheral blood lymphocytes (mesoderm), and hair follicles and saliva (ectoderm) was analyzed with Sanger sequencing (as reported above) and/or the QuantStudio^TM^ 3D Digital PCR (dPCR) system (ThermoFisher Scientific). For dPCR, rare variants were analyzed using Taqman® custom SNPs genotyping assays (Supplementary Methods and Table S2). The rare mutant allele frequency was obtained by dividing the number of copies per microlitre of the mutant allele by the total number of copies per microlitre of the wildtype plus the mutant alleles. The Limit of Detection (LoD) for each Taqman® custom SNPs genotyping assay was assessed by analyzing two genomic DNA samples from healthy subjects. For the Taqman probe c.637C>T (Supplementary Table S2), the LoD was 0.2%, while for the other assays it was 0%. dPCR reactions were performed in triplicate, except for FFPE derived samples that were tested in duplicate for paucity of DNA.

### *APC* second hit analysis

In order to investigate whether an epigenetic, genomic (deletions/duplications), or mutational second hit in the *APC* gene had occurred in patients with proved *APC* mosaicism (*n* = 4), methylation of the *APC* promoter 1A (RefSeq U02509.1), Multiplex Ligation Probe Amplification (MLPA), and *APC* hot spot codons analyses were performed. For methylation analysis, DNA extracted from fresh adenomatous polyps was treated with sodium bisulfite using EZ DNA Methylation-Gold^TM^ (ZymoResearch, Freiburg, Germany), according to the manufacturer’s protocols, and analyzed by bisulfite sequencing. Primers amplifying a sequence located between −327 and −38 from the transcriptional start codon of the promoter 1A and containing 21 CpG dinucleotides were designed using MethPrimer software [22]. MLPA was conducted on DNA extracted from fresh adenomatous polyps and normal mucosa using the SALSA MLPA *APC* probemix (P043, MRC-Holland, Amsterdam, The Netherlands). All data were analyzed using the Coffalyser.Net software (MRC-Holland), which generates a relative probe ratio from the comparison between adenomatous polyps and normal mucosa. A probe ratio below 0.7 or above 1.3 was regarded as indicative of a heterozygous deletion or duplication, respectively. Variants in *APC* gene hot spot codons (1061, 1309, and 1450) were analyzed by Sanger sequencing. Primer sequences and annealing temperatures are reported in Supplementary Table S1A.

### *BRAF*, *KRAS,* and *CTNNB1* analysis

Somatic variants in *BRAF* (c.1799T>A p.(Val600Glu); RefSeq NM_004333.4), *KRAS* (codons 12–13; RefSeq NM_004985.4), and *CTNNB1* (exon 3; RefSeq NM_001904.3) genes were analyzed by Sanger sequencing on available FFPE adenomatous polyps as reported above. Primer sequences and annealing temperatures are reported in Supplementary Table S1B.

### Whole exome sequencing

Whole Exome Sequencing (WES) was performed on genomic DNA isolated from peripheral blood samples of probands and parents (and/or siblings). As DNA from parents of patients P6 and P7 was not available, we conducted WES on probands only in these patients. A DNA library was prepared using Nextera^®^ DNA Library Kit (Illumina Inc. USA) and sequenced on an Illumina HiSeq2000 platform. The obtained reads were aligned to the reference genome GRCh37/hg19 using the Burrow–Wheeler Aligner software [23]. The alignments were stored in the BAM file and processed using GATK and ANNOVAR softwares [24, 25]. Whenever possible, the variants were filtered with the parents/siblings, considering both a dominant and a recessive autosomal inheritance pattern using DeNovoGear and Gemini softwares [26, 27]. Only those variants whose prediction showed a strong effect on gene function were considered for downstream analysis: (1) frameshift variants (insertions/deletions across the coding sequence); (2) variants located within 5 bp from the intron–exon junctions of coding exons (canonical splice-site); and (3) non-synonymous single-nucleotide variants (nonsense and missense). The variants with Minor allele frequency (MAF) ≥0.01 based on data from dbSNP [28], 1000 Genomes Project [29], NHLBI Exome Sequencing project (http://evs.gs.washington.edu/EVS/), Exome Aggregation Consortium [30], and in-house database (264 patients with non-cancer disorders) were filtered out. Regarding the missense variants, only those with possible or probable deleterious effect according to three in-silico prediction tools (PolyPhen2, score ≥0.85; SIFT, score ≤0.5; CADD score ≥3) were selected [31–33]. These variants were further filtered considering the function and expression of the candidate genes. For this reason OMIM [34], Human Protein Atlas [35], COSMIC [36], Colorectal Cancer Atlas [37], and KEGG pathway [38] databases were consulted. The pathogenic relevance of the variants was further explored by evaluating their genetic intolerance to functional variations according to the Residual Variations Intolerance score [39]. All selected variants were validated through Sanger sequencing. For patients P5, P6, and P7, as fresh colonic tissues were not available due to previous surgical procedures (proctocolectomy or colectomy), WES was also performed on DNA from one FFPE adenomatous polyp for each patient to identify somatic *APC* variants. For any candidate variant identified by WES, somatic loss of heterozygosity (LOH) was performed (Supplementary Methods).

All the variant and phenotype data have been submitted to the LOVD database (https://databases.lovd.nl/shared/genes/APC; variants IDs 0000174613-0000174623 and 0000174632).

## Results

### Somatic APC mosaicism in patients with unexplained colorectal adenomatous polyposis

**Fig. 2:**
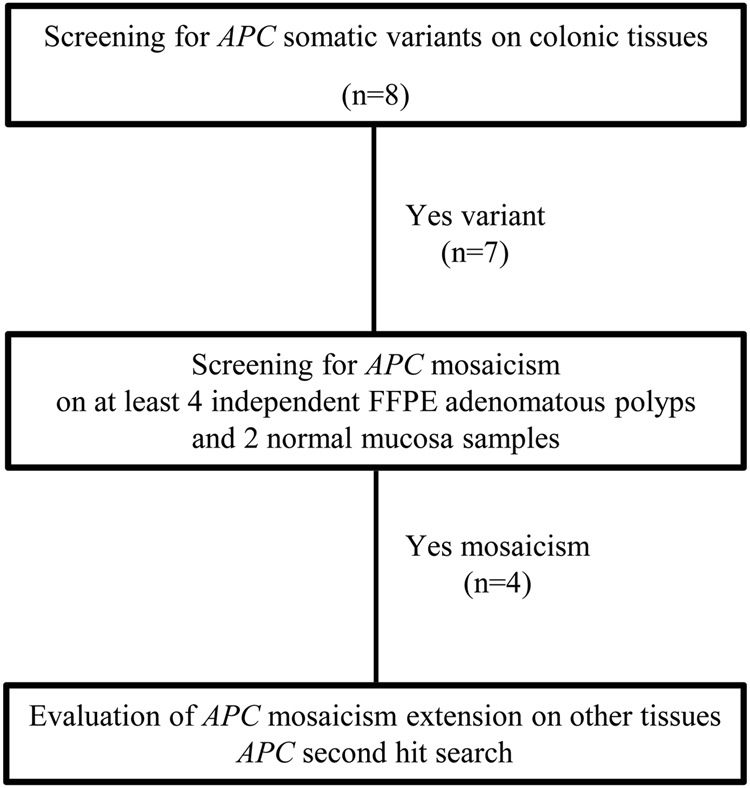
Flowchart of *APC* mosaicism evaluation in enrolled patients. The initial screening of the whole *APC* gene was carried out in one adenomatous polyp sample for each patient, except for patient P1 where two polyps were analyzed

Among the 56 patients with a clinical diagnosis of polyposis, 8 had no causative germline variants in *APC* and/or *MutYH* genes and fulfilled the inclusion criteria. These patients were enrolled (Fig. 1) and evaluated for *APC* mosaicism. A flowchart reporting somatic *APC* variants screening is shown in Fig. 2. The initial investigation of the whole *APC* gene for somatic variants was performed in one polyp sample for each patient, except for patient P1 where two polyps were analyzed.

Seven patients (P1–P7) had a variant in the *APC* gene (Table 2 and Supplementary Table S3). No pathogenic variants in the *APC* gene were found in colonic adenomatous polyps of patient P8 carrier of a germline in-frame deletion in the *APC* gene c.3468_3470delAGA p.(Glu1157del) of unknown significance (VUS). Analysis of multiple adenomatous polyps unveiled a condition of *APC* mosaicism in 50% of patients (P1–P4) with unexplained adenomatous polyposis (Table 2).

**Table 2:** *APC* variants and rare variant allele frequencies in patients with mosaicism

Patient	Variant	Tissue	Localization	Sanger	dPCR (%)
P1	c.637C>T p.(Arg213*)	Fresh NM	n.a.	Wt	0.37
		Fresh Polyp 1	n.a.	Variant	19.2
		Fresh Polyp 2	n.a.	Variant	12.2
		FFPE NM 1	Hepatic flexure/transverse colon	Variant	n.a.
		FFPE NM 2	Transverse colon	Wt	n.a.
		FFPE NM 3	Sigmoid colon	Wt	1.96
		FFPE Polyp 1	Hepatic flexure/transverse colon	Variant	n.a.
		FFPE Polyp 2	Hepatic flexure/transverse colon	Variant	n.a.
		FFPE Polyp 3	Splenic flexure/discending colon	Variant	n.a.
		FFPE Polyp 4	Transverse colon	Variant	n.a.
		FFPE Polyp 5	Transverse colon	Variant	26.5
		FFPE Polyp 6	Sigmoid colon	Variant	27.3
		FFPE Polyp 7	Sigmoid colon	Variant	n.a.
P2	c.2626C>T p.(Arg876*)	Fresh NM	n.a.	Wt	0
		Fresh Polyp 1	n.a.	Variant	21.1
		Fresh Polyp 2	n.a.	Variant	8.9
		FFPE NM 1	Ascending colon/hepatic flexure	Variant	n.a.
		FFPE NM 2	Sigmoid colon	Variant	n.a.
		FFPE NM 3	Sigmoid colon	n.a.	9.7
		FFPE NM 4	Sigmoid colon	n.a.	0
		FFPE Polyp 1	Ascending colon/hepatic flexure	Variant	17.6
		FFPE Polyp 2	Ascending colon/hepatic flexure	Variant	n.a.
		FFPE Polyp 3	Sigmoid colon	Variant	n.a.
		FFPE Polyp 4	Sigmoid colon	Variant	n.a.
		FFPE Polyp 5	Sigmoid colon	Variant	33.4
		FFPE Polyp 6	Sigmoid colon	Variant	n.a.
P3	c.4393_4394delAG p.(Ser1465Trpfs*3)	Fresh NM	n.a.	Wt	0
		Fresh Polyp	n.a.	Variant	18.5
		FFPE NM 1	Sigmoid colon-rectum	Wt	0.9
		FFPE NM 2	Sigmoid colon-rectum	n.a.	4.5
		FFPE Polyp 1	Sigmoid colon-rectum	Variant	n.a.
		FFPE Polyp 2	Sigmoid colon-rectum	Variant	30.8
		FFPE Polyp 3	Sigmoid colon-rectum	Variant	n.a.
		FFPE Polyp 4	Sigmoid colon-rectum	Variant	18.4
		FFPE Polyp 5	Sigmoid colon-rectum	Variant	n.a.
P4	c.3927_3931delAAAGA p.(Glu1309Aspfs*4)	Fresh NM	Ascending colon	Wt	0
		Fresh Polyp	Ascending colon	Variant	26.4
		FFPE NM 1	Ascending colon	Wt	6.6
		FFPE NM 2	Approx 45 cm ab ano	Wt	0
		FFPE Polyp 1	Ascending colon	Variant	26.4
		FFPE Polyp 2	Ascending colon	Variant	n.a.
		FFPE Polyp 3	Hepatic flexure	Variant	24.8
		FFPE Polyp 4	Approx 45 cm ab ano	Variant	18.7

*NM* normal mucosa, *Polyp* adenomatous polyp, *FFPE* Formalin-Fixed Paraffin-Embedded, *n.a.* not analyzed, *Wt* wildtype, *dPCR* digital PCR, *APC* variants description refers to RefSeq NM_000038.5

To establish *APC* mosaicism extent in these patients, further tissues (normal colonic mucosa, blood, hair follicles, and saliva) were analyzed through Sanger sequencing and dPCR. Importantly, dPCR proved to be more sensitive compared to Sanger sequencing. Indeed, dPCR allowed us to identify *APC* variants in fresh and FFPE normal mucosa samples of patient P1 and in one normal mucosa sample of patients P2, P3, and P4, despite being present at low frequency. In addition, while mosaicism was confined to the colon in three out of four patients (P1, P2, and P4), in one patient (P3) an extension to the duodenum (52%) and saliva was also found by dPCR (0.25%). We also excluded the involvement of other known predisposing colorectal polyposis genes, including *POLE*, *POLD1*, *NTHL1,* and *MSH3*, by WES on DNA from peripheral blood.

### APC second hit in mosaic patients

To identify whether patients with *APC* mosaicism also harbored a second hit in the *APC* gene, we first analyzed hot spot codons for somatic inactivating variants, finding a second hit in three out of four patients. In patient P1, we identified a frameshift variant affecting the hot spot codon 1309 (c.3927_3931delAAAGA p.(Glu1309Aspfs*4)) in one FFPE adenoma sample and another frameshift variant (c.4187_4188delTT p.(Phe1396*)) in another two different FFPE adenoma samples. In patient P2, a variant affecting the hot spot codon 1450 (c.4348C>T p.(Arg1450*)) was found in 1/6 FFPE adenoma samples, while patient P3 harbored a second hit in the codon 1027 (c.3081_3085delinsGAG p.(Tyr1027*)) in 2/5 FFPE adenoma samples. In patient P4, no hot spot mutational events were found. Neither aberrant methylation of promoter 1A nor duplications/deletions in the *APC* gene coding region were found in any patient.

### Variants in known CRC predisposing genes

To evaluate the presence of additional somatic mutational events in other genes critically involved in CRC development, we analyzed *BRAF*, *KRAS*, and *CTNNB1*. All patients were wildtype for *BRAF* and *CTNNB1*. Patient P2 showed two heterozygous variants in *KRAS* (c.35G>A p.(Gly12Asp) and c.38G>A p.(Gly13Asp)) in different adenomatous polyps, while patients P5 and P7, both had a heterozygous KRAS variant in one polyp (P5: c.34G>A p.(Gly12Ser); P7: c.35G>A p.(Gly12Asp)).

### WES analysis and oligogenic inheritance in rare variants

**Fig. 3:**
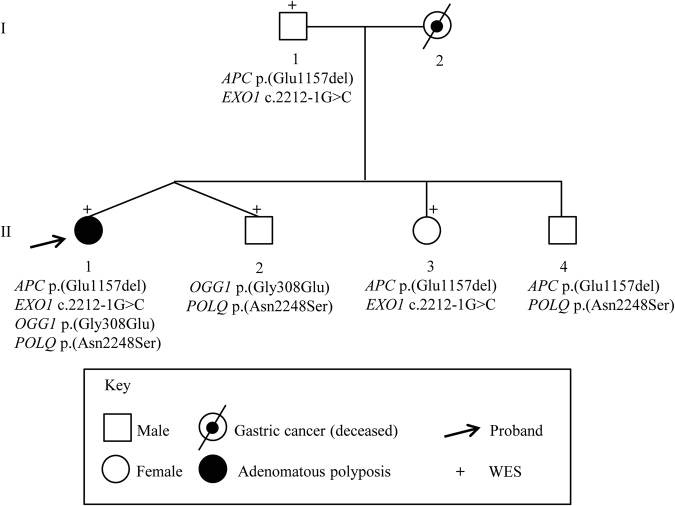
Pedigree of patient P8. Variants are reported in proband and in family members. All relatives, except the father (I.1), underwent colonoscopy. Variants description refers to the following reference sequences: *APC* NM_000038.5; *OGG1* NM_016829.2; *EXO1* NM_130398.3 NG_029100.1; and *POLQ* NM_199420.3

Enrolled patients had no family history of adenomatous polyposis, suggesting that *APC* somatic mosaicism, de novo variants, biallelic variants (recessive inheritance), or polygenic inheritance could explain their clinical phenotype. Thus, to identify genetic defects occurring in patients without *APC* mosaicism or causative *APC* variant (*n* = 4) or to identify potential additional pathogenic variants in patients with *APC* mosaicism, WES was performed on DNA from peripheral blood. For patients P1–P7, no additional or causative polyposis variants were found. Intriguingly, WES analysis of patient P8, a carrier of the VUS c.3468_3470delAGA p.(Glu1157del) (rs386833391; MAF not available) in the *APC* gene, showed a missense variant c.923G>A p.(Gly308Glu) (rs113561019) in the *8-Oxoguanine DNA glycosilase* (*OGG1* RefSeq NM_016829.2), a splicing variant c.2212–1G>C (rs4150000) in the *Exonuclease 1* (*EXO1* RefSeq NM_130398.3 NG_029100.1), and a missense variant c.6743A>G p.(Asn2248Ser) (rs376729696) in the *DNA Polymerase theta* (*POLQ* RefSeq NM_199420.3) genes. The results of the prediction tools for the missense and splicing variants and MAF values are reported in Supplementary Table S4. All identified variants were heterozygous and no LOH was found. However, since we performed only Sanger sequencing for the regions containing the variants, we cannot rule out other kinds of second hits. Importantly, segregation analysis showed that the co-occurrence of two of these variants was not sufficient to cause the phenotype being present in all unaffected members of the family (Fig. 3). Thus, we can hypothesize that the combination of these four variants could be responsible for polyposis development in patient P8.

## Discussion

A considerable proportion of colorectal adenomatous polyposis cases remain genetically unsolved. In this study, we aimed to identify the genetic cause of adenomatous polyposis in patients with no germline variants in known predisposing genes. *APC* mosaicism is emerging as an important mechanism for polyposis onset [10–12, 40]. Noteworthy, we found somatic *APC* mosaicism in 50% of the enrolled patients. We believe that although our restrictive inclusion criteria (no family history of polyposis, age at diagnosis, and number of adenomatous polyps) reduced the number of eligible patients, they allowed us to efficiently intercept *APC* mosaic patients. Indeed, previously published studies found a lower percentage of *APC* mosaicism [10–12], and only one recent study identified an *APC* mosaicism rate corresponding to ~50% [41]. It is to note that the percentage of identification of mosaic cases depends on the inclusion criteria of the study and, if mainly attenuated polyposis cases are included, as in this study, the detection rate seems to be higher.

Mosaicism extension depends on the time when the variant occurs during embryogenesis. Interestingly, in our study, mosaicism was confined to the colon in three patients, suggesting that the first mutational event affected the endoderm. Conversely, in one patient with a more complex clinical phenotype (>150 colonic adenomas, duodenal adenomas, bilateral sensorineural hearing loss, and diabetes mellitus type II), mosaicism was also extended to the duodenum and saliva, involving both the endodermal and ectodermal layers. In addition, we found no variants in peripheral blood and hair follicles in any patient. Moreover, we checked the positions of *APC* mosaic variants in WES data obtained from leukocyte DNA and we did not find any variant alleles (based on a coverage of 45–147 reads).

In this study the *APC* mosaicism search was performed in a large number of colonic samples and additional tissues. Interestingly, the *APC* variants could not be detected in some normal mucosa colonic samples, as reported also by Jansen and colleagues [41]. We speculate that this pattern might suggest a condition of intra-organic mosaicism in the colon. Moreover, we believe that the analysis of multiple adenomatous polyps should be recommended as a future direction for more definitive studies on mosaicism identification. Noteworthy, our data highlight the importance of using highly sensitive technologies, such as dPCR, in order to increase the likelihood of detecting *APC* mosaicism extension. In particular, if only gastrointestinal tissues are affected by *APC* mosaicism, but not peripheral blood or ectodermal-derived tissues, the risk of transmission to the offspring is low.

Intriguingly, WES analysis allowed us to identify rare variants in *OGG1*, *EXO1*, and *POLQ* genes in a patient carrying the germline *APC* VUS c.3468_3470delAGA p.(Glu1157del). Importantly, we described, for the first time, that the combination of these four variants represents an oligogenic inheritance pattern that may explain colorectal polyposis in this patient. These variants have already been described [42–46]. *OGG1* and *EXO1* are involved in the BER and MMR pathways and could act as low-penetrance alleles contributing to adenomatous polyposis and CRC progression [43–46], while *POLQ* is implicated both in maintaining genomic stability and BER [47, 48]. The combined effect of variants in *APC*, *EXO1*, and in the endonuclease *FEN1* was previously found to promote gastrointestinal carcinogenesis in mice [49]. In addition, a combination of germline variants in *OGG1* and *MutYH* genes has been reported as a model of digenic inheritance for early colorectal adenomas and cancer development in one patient [44].

Although our results confirm the relevance of *APC* gene mosaicism as an underlying cause of colorectal polyposis, we acknowledge that this study has some limitations. First, the number of patients is small. Second, we cannot exclude the remote possibility of additional causative variants in other genes not investigated in this study. Third, due to the paucity of available material, *APC* second hit analysis involved hot spot codons only. Fourth, since the initial analysis of the whole *APC* gene for somatic variants was performed only in one adenomatous polyp for each patient (except for patient P1), we might have missed *APC* mosaicism in the other four patients because the “variant of interest” could be below the detection threshold of Sanger sequencing/WES in the polyp analyzed, but maybe in other polyps it would be detectable.

In conclusion, our study provides new insights for the genetic characterization and screening of patients with unexplained adenomatous polyposis. In view of our findings, for a more accurate assessment of patients carrying *APC* mosaicism and of their offspring, we highly recommend the collection and testing of multiple adenomas, normal-appearing colonic mucosa, and other biological samples. We believe that larger cohorts and more studies are needed to explore the percentage of *APC* mosaic cases more comprehensively. Finally, we propose a new oligogenic inheritance model to explain an unsolved case of polyposis.

## Electronic supplementary material

Supplementary Material and Methods

Supplementary Table S1A and S1B

Supplementary Table S2

Supplementary Table S3

Supplementary Table S4
