# Supplementary material for: Somatic APC mosaicism and oligogenic inheritance in genetically unsolved colorectal adenomatous polyposis patients
Source: Eur J Hum Genet. 2018 Jan 24;26(3):387–95. doi: 10.1038/s41431-017-0086-y (PMC5839046; doi:10.1038/s41431-017-0086-y)
Supplement: Supplementary file 1 [file 41431_2017_86_MOESM5_ESM.pdf]

Supplementary Table S4. MAF values and prediction for the missense and splicing variants

Gene	Variant	RefSeq	dbSNP ID	MAF (ExAc)	PolyPhen-2	SIFT	Scaled CADD score	Human splicing finder
OGG1	c.923G>A	NM_016829.2	rs113561019	A=0.0041/500	probably damaging	damaging	28.7	/
EXO1	c.2212-1G>C	NM_130398.3<br>NG_029100.1	rs4150000	C=0.0019/214	/	/	16.82	Alteration of the WT acceptor site,<br>most probably affecting splicing
POLQ	c.6743A>G	NM_199420.3	rs376729696	C=0.0001/16	probably damaging	damaging	24.4	/

Legend: MAF: Minor Allele Frequency; CADD: Combined Annotation Dependent Depletion;
Scaled CADD score threshold: ≥10 mildly damaging; ≥20 moderately damaging; ≥30 strongly damaging.
