# Supplementary material for: Somatic APC mosaicism and oligogenic inheritance in genetically unsolved colorectal adenomatous polyposis patients
Source: Eur J Hum Genet. 2018 Jan 24;26(3):387–95. doi: 10.1038/s41431-017-0086-y (PMC5839046; doi:10.1038/s41431-017-0086-y)
Supplement: Supplementary file 2 [file 41431_2017_86_MOESM1_ESM.doc]

**SUPPLEMENTARY METHODS**

**Sanger sequencing**

For Sanger sequencing 20 ng of genomic DNA were amplified with KAPA 2G Fast HotStart Ready Mix (Kapabiosystems, Wilmington, USA) and the following PCR conditions: denaturation for 3 minutes at 95°C, followed by 40 cycles of denaturation for 15 seconds at 95°C, annealing for 15 seconds and extension for 5 seconds at 72°C, and final extension for 1 minute at 72°C. Sequencing was performed on PCR products purified with ExoSAP-IT (USB, Cleveland, Ohio, USA) using ABI Prism Big Dye Terminator Cycle Sequencing kit v.1.1 (Thermofisher Scientific); then products were purified with Montage SEQ96 Sequencing Reaction Cleanup Kit (Millipore Corporation, Billerica, USA) and analyzed on the ABI Prism 3730 DNA Analyzer (Thermofisher Scientific).

**dPCR**

dPCR reactions were prepared in a final volume of 15 μl mixing 7.5 μl of 2X QuantStudio 3D Digital PCR Master Mix v2 (ThermoFisher Scientific), 0.375 μl of 40X Taqman® assay probemix (ThermoFisher Scientific), target DNA (diluted in order to obtain about 1045 copies/μl or 3.45 ng/μl) and water. The reaction was subsequently loaded on the QuantStudio 3D Digital PCR Chip v2 (ThermoFisher Scientific) and PCR performed on the ProFlex^TM^ 2X Flat PCR System (ThermoFisher Scientific) using the following cycling conditions: denaturation for 10 minutes at 96°C followed by 39-52 cycles of annealing/extension for 2 minutes at 60-62°C, denaturation for 30 seconds at 98°C and one cycle of final extension for 2 minutes at 60°C. Fluorescence signals were measured with the QuantStudio 3D Digital PCR Instrument (ThermoFisher Scientific) and the calculation of the absolute number of positive events for a given channel (FAM or VIC) was performed by the on-line QuantStudio 3D Analysis Suite^TM^ Software release 3.1.1 (ThermoFisher Scientific).

**Loss of heterozygosity analysis**

Loss of heterozygosity **(**LOH) was assessed by Sanger sequencing using the conditions described above. The analysis was conducted on peripheral blood and FFPE adenomatous polyp DNA samples. Primer sequences are listed in Supplementary Table S1B.
