# Supplementary material for: Somatic APC mosaicism and oligogenic inheritance in genetically unsolved colorectal adenomatous polyposis patients
Source: Eur J Hum Genet. 2018 Jan 24;26(3):387–95. doi: 10.1038/s41431-017-0086-y (PMC5839046; doi:10.1038/s41431-017-0086-y)
Supplement: Supplementary file 3 [file 41431_2017_86_MOESM2_ESM.doc]

**Supplementary Table S1A. *APC* Primers**

| **Amplicon** | **Forward Primer (5’-3’)** | **Reverse Primer (5’-3’)** | **A.T.** |
| --- | --- | --- | --- |
| Promoter 1A | TGGGAATTTAAGCATCACCT  chr5: 112737060_112737079* | ACACGCATAGTAAAGAGTCG  chr5: 112737592_112737611* | 60°C |
| Promoter 1A | TTATTGTTTCTCTGTGCTGC  chr5: 112737484_112737503* | AGACTCCCCTTAACACTTCT  chr5: 112738155_112738174* | 60°C |
| Promoter 1B | GTGAGACAAACAAGGATTTCC  chr5:112707038_112707058* | GAGGTGGGAAGACTACAATG  chr5:112707499_112707518* | 60°C |
| Promoter 1B | TGCGGAATAATGGACTAGTG  chr5:112707267_112707286* | GATGGGGAAGCGACTAAAAT  chr5:112708194_112708213* | 60°C |
| Exon 4 | ACACAATTCTTCTTAAACGTCT | TAAGTAAAGTGTCTTACCTCAA | 60°C |
| Exon 5 | CTCAGCATACTTAAATGTCAAGA | AACTGATACCAACACCCAAAT | 60°C |
| Exon 6 | TCAGTCATGTATATTTGTGGTT | GTAAGTGCAATCAATAACATGC | 60°C |
| Exon 7 | TCTTTTAAGGATGATTACCAGT | TATTCAGGCCTAAAGTTGGG | 60°C |
| Exon 8 | GACTGACGTATTTGCTTATTCA | TCAGCAGCCATGATAAATCTT | 60°C |
| Exon 9 | TGAACTGACCCCAATTTGTTA | ACGGTATTTAAATTCTCTGAAAG | 60°C |
| Exon 10 | CCATGCCTTTATCAGTCTGTA | TGGAAATATGCATTCAGGACT | 60°C |
| Exon 11 | CAGACACTTCATTTGGAGTACC | AGTAGAGATGGGGTTTTGCC | 60°C |
| Exon 12 | TGGTGATGATACATAGATTTTGA | AGTAGCACAAATGGCTGATAT | 60°C |
| Exon 13 | TGTTGATCCACTAAAATTCCG | GTTGGTGGCCTTATATCCTAA | 60°C |
| Exon 14 | TGTAGCTTATAATTCTAAAGGCA | AAAGATAAGCGAATGTGAAGC | 60°C |
| Exon 15 | AGGAAGAACAGATAGCAAAGA | GAGCTGAGATTGCACAACT | 60°C |
| Exon 16 | GATAGGATTACAGGCGTGAG | ACACAGGTAAGAAATTAGGA | 60°C |
| Exon 17 | TGGAAAGTTCTTAATTTACCAGT | AGAGTTAGTAGTTATCTTTTCAC | 60°C |
| Exon 18 | AGAACAAAAGGAGATGTGGAA | ACCTATTATCATCATGTCGA | 60°C |
|  | TCAAGTGGAAGTGAATCTGAT | TTCAGCACCATTTGTAGCAC | 60°C |
|  | AAGGCATCTCATCGTAGTAAG | TGACCTCTTTTACCATAACCA | 60°C |
|  | TAAAGAAAACCAAGTATCCGC | CTAGGGCTTGGGTTGTAATTA | 60°C |
|  | TTCTATAGTGGAACGTACCCC | CCCTCTAACAAGAATCAAACC | 60°C |
|  | GTCGGAAAATTCAAATAGGACA | CGTTCACTATAATTGGTAGGC | 60°C |
|  | GAGTGGGTTCTAATCATGGAA | CCTGGCTGATTCTGAAGATAA | 60°C |
|  | AAAGATTGGAACTAGGTCAGC | GTGGTGGAGGTGTTTTACTT | 60°C |
|  | ACTTGATAGTTTTGAGAGTCGT | CTGTTTTGTGATGGTAGAAGTT | 60°C |
|  | TATTATTTCTGCCATGCCAAC | TGTGGTATAGGTTTTACTGGT | 60°C |
|  | AAGCAGAGGAAGGTGATATTC | GAAAAGTGGATTGTTTCTGAAG | 60°C |
|  | AACTAACCTCCAACCAACAAT | ACTTACTATGGAATTTGCACC | 60°C |
|  | TCTGACACTTGATTTGAAAGA | TAGAAGGTGCTTTACTTGACC | 60°C |
|  | CTCCAAAAGCCCTAGTGAAG | GTCTAGATGATGGAGAAAGAGA | 60°C |
| Codon 1061 | GTTATGGTCAATACCCAGCCG | GGATAAGTTGTACTTTGATTC | 56°C |
| Codon 1309 | CAGACTTATTGTGTAGAAGAT | TTGGTTCTAGGGTGTGTGAC | 56°C |
| Codon 1450 | GTTTAGCAGATGTACTTCTGT | TCTCTCTCTTTTCAGCAGTAG | 52°C |
| Bisulfite sequencing promoter 1A | GTTATTAATTTTTTTGTTTGTTGGG | ACACCTCCATTCTATCTCCAATAAC | 57°C |

Legend: A.T.: annealing temperature; *APC* exons are numbered as in the RefSeq NG_008481.4. *APC* Promoter 1A Refseq is U02509.1; *APC* Promoter 1B RefSeq is D13981.1; * reference genome GRCh37/hg19.

**Supplementary Table S1B. Primers**

| **Gene** | **RefSeq** | **Forward Primer (5’-3’)** | **Reverse Primer (5’-3’)** | **A.T.** |
| --- | --- | --- | --- | --- |
| *BRAF* | NG_007873.3 | TGCTTGCTCTGATAGGAAAATGA | TGGATCCAGACAACTGTTCAAA | 57°C |
| *KRAS* | NG_007524.1 | AAGGCCTGCTGAAAATGACTG | AAAGAATGGTCCTGCACCAG | 57°C |
| *CTNNB1* | NG_013302.2 | TCAATGGGTCATATCACAGA | TCTCAAAACTGCATTCTGAC | 57°C |
| *EXO1* | NG_029100.1 | GCGACAGAGTGAGAGTCCAT | GCTTTCTCTTCTGGATGCTTG | 62°C |
| *OGG1* | NG_012106.1 | AGTCACCTCTCCCTCAGACC | GACAGGGGTGGCTGGAGA | 64°C |
| *POLQ* | NC_000003.11 (121150273.. 121264853)* | TGGGATGGTCTTGTACTTATT | AGGACAGAAATGGGTATGTC | 60°C |

Legend: * reference genome GRCh37/hg19; A.T.: annealing temperature
