# Supplementary material for: Somatic APC mosaicism and oligogenic inheritance in genetically unsolved colorectal adenomatous polyposis patients
Source: Eur J Hum Genet. 2018 Jan 24;26(3):387–95. doi: 10.1038/s41431-017-0086-y (PMC5839046; doi:10.1038/s41431-017-0086-y)
Supplement: Supplementary file 4 [file 41431_2017_86_MOESM3_ESM.doc]

**Supplementary Table S2. Taqman *APC* genotyping probes and primers**

| ***APC* variant** | **Forward Primer (5’-3’)** | **Reverse Primer (5’-3’)** | **VIC** | **FAM** |
| --- | --- | --- | --- | --- |
| c.637C>T | CAACTAGGTACCTGCCAGGATATG | AACAGAGCTGTAATTCATTTTATTCCTAATAGCT | CTGTGCTCGTTTTTC | TGTGCTCATTTTTC |
| c.2626C>T | CAGCAACAGAAAATCCAGGAACTTC | GGCTGCAGTGGTGGAGATC | TGCAAACCTCGCTTTGA | TGCAAACCTCACTTTGA |
| c.4393_4394delAG | CTCAAACCAAGCGAGAAGTACCTAA | CCTCTGAACTGCAGCATTTACTG | AAAAGAGAGAGAGTGGACCT | TGAAAAGAGAGAGTGGACCT |
| c.3927_3931delAAAGA | AGATTCTGCTAATACCCTGCAAATAGC | AACTTCGCTCACAGGATCTTCAG | CCTAGTTCCAATCTTTTCTTTTA | ACCTAGTTCCAATCTTTTA |

Legend: VIC: wild-type allele probe; FAM: variant allele probe; *APC* variants description refers to RefSeq: NM_000038.5
