# Supplementary material for: Somatic APC mosaicism and oligogenic inheritance in genetically unsolved colorectal adenomatous polyposis patients
Source: Eur J Hum Genet. 2018 Jan 24;26(3):387–95. doi: 10.1038/s41431-017-0086-y (PMC5839046; doi:10.1038/s41431-017-0086-y)
Supplement: Supplementary file 5 [file 41431_2017_86_MOESM4_ESM.doc]

**Supplementary Table S3. *APC* variants** in patients without mosaicism

| **Patient** | ***APC* variant** | **COSMIC ID** |
| --- | --- | --- |
| P5 | c.4132C>T p.(Gln1378*) | COSM18862 |
| P6 | c.4233delT p.(Ser1411Argfs*4) | COSM18948 |
| P7 | c.694C>T p.(Arg232*) | COSM13130 |

Legend: *APC* variants description refers to RefSeq NM_000038.5
